# Effects of Alpha Particle Exposure on Genetic Stability and Morphogenesis in *Drosophila melanogaster*

**DOI:** 10.3390/biology15100789

**Published:** 2026-05-15

**Authors:** Zarema Biyasheva, Yuliya Zaripova, Anna Lovinskaya, Vyacheslav Dyachkov, Alexandr Yushkov

**Affiliations:** 1 National Nanotechnology Laboratory of Open Type (NNLOT), Farabi University, Almaty 054000, Kazakhstan; zarema.biyasheva@kaznu.edu.kz (Z.B.); anna.lovinskaya@kaznu.edu.kz (A.L.); aleksandr.yushkov@kaznu.edu.kz (A.Y.); 2 Department of Nuclear Physics, Voronezh State University, Voronezh 394018, Russia; lnirp206@gmail.com

**Keywords:** *Drosophila melanogaster*, alpha radiation, radon, morphological abnormalities, teratogenic effects, radiation biology, ecological genetics

## Abstract

The study of genetic effects induced by low-dose alpha radiation associated with radon and its decay progeny is critically important for assessing radiation risks in regions with elevated natural background levels. The aim of this study was to evaluate the mutagenic effects (in germline cells) and teratogenic effects (in somatic tissues) of alpha radiation using the *D. melanogaster* model. To differentiate between these effects, teratogenic outcomes were analyzed in directly exposed individuals (phenotypic analysis of adults that developed from irradiated larvae), whereas mutagenic effects were assessed in the progeny of irradiated flies. Larvae and adult flies were exposed to calibrated alpha-particle sources with energies ranging from 4.8 to 7.7 MeV and absorbed doses of 1.90–44.96 mGy. The results demonstrated a statistically significant increase in the frequency of morphological abnormalities in the exposed groups, including melanotic masses and deformities of the wings, thorax, and tergites. Under 72 h exposure, a strong correlation between absorbed dose and abnormality frequency was observed (r = 0.98). In the reporter system, induction of GFP expression was detected in imaginal discs at doses above 10 mGy, indicating threshold activation of the cellular stress response. The obtained data demonstrate that chronic low-dose α-irradiation leads to an increased frequency of morphological abnormalities (indirect phenotypic manifestations of compromised genetic stability) in *D. melanogaster*, with the most pronounced effects observed at the level of morphogenesis. The high sensitivity of the applied test systems was confirmed, supporting the use of *D. melanogaster* as a bioindicator for ecogenetic monitoring of radon-prone areas, including regions of Kazakhstan.

## Simple Summary

This study investigated how low-dose alpha radiation, similar to that from natural radon gas, affects genetic stability and development using fruit flies (*Drosophila melanogaster*). It enables us to study fundamental mechanisms of radiation-induced genome instability in both somatic and germline cells. Larvae and adult flies were exposed to low doses of alpha radiation (1.90–44.96 mGy). In larvae, direct developmental abnormalities (including malformed wings, body deformities, and melanotic spots) were assessed, whereas in the offspring of irradiated adults, inherited defects were examined. Alpha radiation induced a statistically significant increase in the frequency of developmental abnormalities, including wing malformations, body deformities, and melanotic lesions. These effects were dose- and time-dependent, becoming more pronounced with increasing radiation dose and exposure duration. Using a fluorescent reporter system, it was shown that radiation doses above 10 mGy triggered cellular stress responses in fly tissues. Notably, a clear dose–response relationship was observed after 72 h of exposure, indicating that even low levels of alpha radiation can induce measurable biological effects. The high sensitivity of *D. melanogaster* supports its use as a valuable model for ecogenetic monitoring of radiation risks in radon-prone regions; however, these results should be interpreted within the framework of genotoxicity assessment rather than as a direct model of oncogenic risk in humans.

## 1. Introduction

In the 21st century, cancer remains one of the major social, healthcare, and economic burdens, particularly in the context of identifying and preventing risk factors contributing to cancer development [1,2,3,4]. Among the numerous factors influencing carcinogenesis, increasing attention is being paid to environmental and radiation-related influences. In particular, considerable interest has been directed toward investigating genetic and epigenetic alterations induced by exposure to radon and its decay progeny (DP), which constitute a major source of background radiation exposure in the general population [5,6,7].

Ecogenetic monitoring of ionizing radiation represents an important area of research for assessing radiation risks in radon-prone areas. In light of the significant impact of radon on human health—particularly its well-established association with lung cancer [5,6,7,8,9,10] and its potential link to skin cancer [11,12,13]—the investigation of genetic effects induced by exposure to different radon levels is of considerable interest. Alpha particles generated during radon decay, particularly from the isotopes ^218^Po and ^214^Po, are capable of penetrating the skin to depths of approximately 47 to 70 μm, respectively, enabling them to reach the basal layer of the epidermis in certain body regions [14,15,16]. Theoretical estimates suggest that up to 0.7% of skin cancer cases may be attributable to indoor radon exposure [17]. Importantly, when extrapolating these findings to humans, where alpha particles rarely reach germline cells because of their limited penetration depth, the most relevant effects are the direct somatic effects on irradiated tissues. Therefore, in the present study, we distinguish between: (1) teratogenic effects, assessed directly in adults that developed from irradiated larvae; and (2) potential heritable mutagenic effects, evaluated in the progeny of irradiated adults. It is important to emphasize that the morphological abnormalities recorded in this study represent non-heritable phenotypic anomalies; however, an increased frequency of such abnormalities in the progeny may serve as an indirect indicator of genetic instability.

Radiation-induced melanogenesis is a complex process involving both direct DNA damage and the activation of oxidative stress pathways. Unlike ultraviolet radiation, which primarily affects superficial layers, α-particles emitted by radon decay progeny are characterized by high linear energy transfer (LET), leading to the formation of clustered DNA damage in melanocytes of the basal layer [16]. Studies indicate that radiation activates key signaling pathways—including Ras/MAPK, JAK/STAT, Hippo, Notch, and JNK—which regulate melanocyte proliferation and eumelanin synthesis, potentially promoting cellular transformation. In *D. melanogaster*, the formation of melanotic spots (“pseudotumors” or melanotic tumors) is considered a consequence of dysregulation of the immune response and hemocyte proliferation, often associated with genetic instability induced by external stressors, including high-energy radiation [18]. Notably, *D. melanogaster* serves as a model of inflammation-mediated carcinogenesis rather than direct melanogenesis. In both larvae and adults, commonly observed phenotypes include melanotic masses (melanotic tumors), hemocyte hyperplasia, nodules in the hemolymph, and defects in differentiation [19]. This makes *D. melanogaster* a suitable model for studying radiation-induced disturbances in morphogenesis relevant to melanoma-like processes.

The biological effects of radon are primarily mediated by the induction of DNA double-strand breaks, leading to genomic instability, chromosomal aberrations, and mutations in tumor suppressor genes (*TP53*, *RB1*) [20,21], oncogenes (*KRAS*, *EGFR*) [22,23], and DNA repair genes (*BRCA1*, *BRCA2*, *ATM*) [24,25]. Individual radiosensitivity, determined by genetic variability in DNA repair systems, underscores the importance of identifying suitable model systems for risk assessment.

As a model system, *D. melanogaster* was selected due to its numerous advantages: approximately 75% of human disease-associated genes have functional homologs in *D. melanogaster* [26]; a short life cycle (approximately 10 days); high fecundity; and ease of maintenance. *D. melanogaster* is widely used for modeling various types of cancer [27,28] and in radiobiological research, dating back to the pioneering work of Muller [29,30,31,32].

Despite decades of research, the majority of radiobiological studies have been conducted using gamma and beta radiation [33,34,35]. In contrast, the effects of highly ionizing α-particles—accounting for more than 50% of the dose from natural radiation sources and increasingly applied in nuclear medicine—remain insufficiently studied. Of particular interest is the evaluation of the linear no-threshold (LNT) model, which postulates that even low doses of radiation are capable of inducing genetic damage [36,37].

Kazakhstan, which ranks second globally in uranium reserves, is characterized by an elevated natural radiation background of 3.1 mSv/year [38,39], exceeding the global average of 2.4 mSv/year [40]. The Almaty region, located in a seismically active zone with numerous tectonic faults [41], is classified as a radon-prone area. This underscores the regional relevance of the present study.

The aim of the present study is to provide a comprehensive assessment of the mutagenic and teratogenic effects of low-dose α-radiation using the *D. melanogaster* model, employing classical genetic assays, direct phenotypic analysis of irradiated individuals, and a GFP reporter system. The results obtained are expected to improve the accuracy of radiation risk assessment and may contribute to the development of effective radiation safety strategies. Given that a substantial proportion of the population remains unaware of the potential hazards associated with radon and does not implement measures to reduce its indoor concentration, the findings of this study may also support the advancement of personalized medicine approaches and the improvement of indoor radiation protection strategies aimed at reducing the impact of environmental carcinogens on public health.

## 2. Materials and Methods

### 2.1. Drosophila Melanogaster Stocks and Husbandry

*Drosophila melanogaster* stocks were maintained on a standard semolina-based medium at 25 °C under a 12 h light/dark cycle. Fly stocks were obtained from the Bloomington Drosophila Stock Center (Bloomington, IN, USA) and the collection of the Institute of Molecular and Cellular Biology (Novosibirsk, Russia). The following strains were used in this study: *Muller-5 (Basc)* (Bloomington Drosophila Stock Center (BDSC) #103238); *Oregon-R* (BDSC #2376); *P{w[+mC] = UAS-GFP.nls}8* (BDSC #4776); *P{GAL4-ninaE.GMR}12* (BDSC #1104); *1-112 (C(1)DX, y w f/y ct v f)* (Ilinsky et al., 2013 [42]); *EP-2 (C(1;Y)2, y[+]/0/C(1)RM, y[1] v[1])* (BDSC stock #2486).

### 2.2. Genetic Test Systems

The investigation of the mutagenic and teratogenic effects of alpha radiation was conducted using the *D. melanogaster* model across five distinct test systems to assess the frequency of morphological abnormalities. During all manipulations, flies were subjected to brief anesthesia with diethyl ether in order to minimize stress and potential harm.

#### 2.2.1. Classical *Muller-5 (Basc)* Test

**Figure 1:**
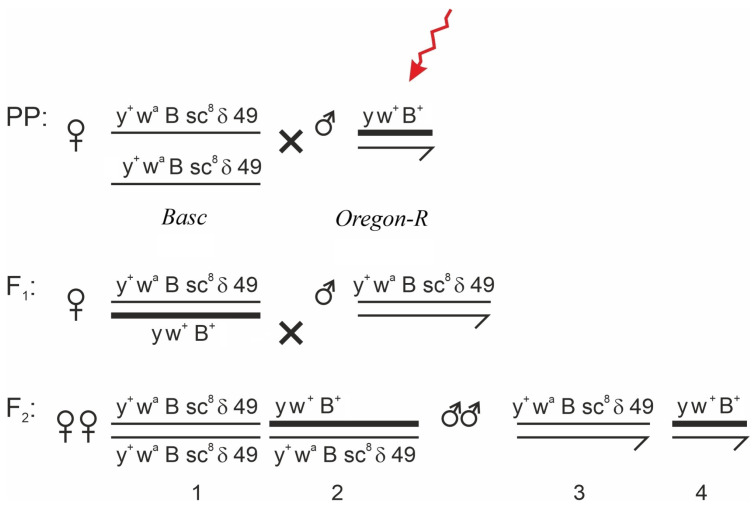
Crossing scheme of *Basc* females with *Oregon-R* males. The arrow indicates the irradiated X chromosome. The F_2_ generation was obtained by intercrossing F_1_ individuals. Morphological abnormalities were identified in F_2_ by analyzing the phenotypes of adult flies carrying the irradiated X chromosome (2 and 4) as indicated in the crossing scheme.

To detect X-linked visible morphological abnormalities transmitted through germline cells, the *Muller-5 (Basc)* method was employed [43,44,45]. The Basc balancer chromosome carries multiple inversions that suppress recombination, as well as a semidominant Bar eye marker, and a recessive *w^a^* (apricot eye) marker, which allows for the easy identification of non-recombinant progeny. The crossing scheme is shown in Figure 1. Irradiated males of the *Oregon-R* line were crossed with virgin females of the *Muller-5 (M-5)* line (3–4 days old). In the F_1_ generation, females carrying the irradiated X chromosome (from the father) and the balancer X chromosome (from the mother) were selected and individually crossed with *M-5* males. In the F_2_ generation, both females and males that inherited the irradiated X chromosome were analyzed for the presence of visible morphological abnormalities. This scheme enables the detection of morphological abnormalities arising in the irradiated paternal X chromosome. Crosses to obtain the F_1_ generation were performed in mass cultures, whereas F_2_ crosses were performed individually. Flies older than 5 days were not used in the experiment, as older individuals tend to accumulate genetic alterations (mutations) that may bias the results [46].

#### 2.2.2. Short-Term Test Systems with Attached X Chromosomes and Attached XY Chromosomes

**Figure 2:**
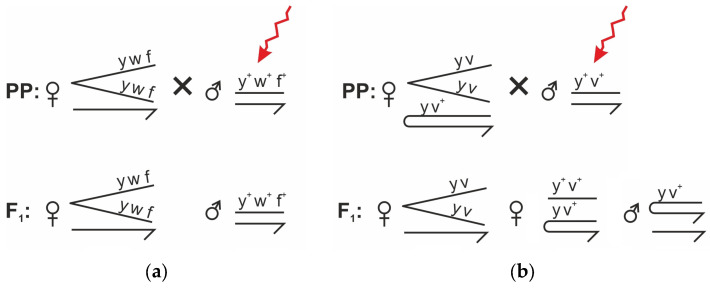
Schematic representation of genetic crosses between *1-112* females (**a**), carrying attached X chromosomes, and *EP-2* females (**b**), carrying attached X and attached XY chromosomes, with *Oregon-R* males. Only viable F_1_ genotypes are shown, as these were used for visual phenotypic analysis.

To detect morphological abnormalities in the first generation (F_1_), irradiated wild-type males (*Oregon-R* strain) were crossed with females from two strains carrying chromosomal rearrangements: *1-112* and *EP-2*. In these crosses, the visible abnormalities identified in the F_1_ offspring are inherited through the germline, as the F_1_ individuals themselves were not directly irradiated. Within the same test systems, teratogenic effects were also investigated following larval irradiation—that is, the directly exposed individuals were analyzed (adults that developed from irradiated larvae). The crossing scheme is presented in Figure 2.

#### 2.2.3. Reporter System Based on Fluorescent Proteins

**Figure 3:**
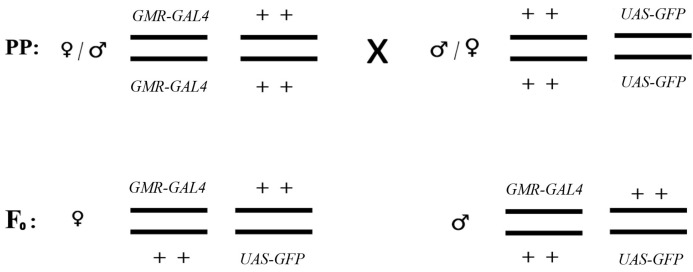
Crossing scheme of the *GMR-GAL4* and *UAS-GFP* genetic lines. PP—parental generation; F_0_—first generation; ♀ and ♂ indicate females and males, respectively.

To assess the teratogenic effects of alpha radiation, a genetic construct-based approach using fluorescent reporter genes was employed. For this purpose, two lines were crossed: the *UAS-GFP* line (carrying the gene encoding green fluorescent protein, GFP) and the *GMR-GAL4* line (expressing the GAL4 driver). The crossing scheme is presented in Figure 3. Specifically, larvae of the F_0_ generation (harboring the *GMR-GAL4* and *UAS-GFP* constructs) were irradiated with calibrated α-particle sources in accordance with the protocol described in Section 2.3, after which imaginal discs were dissected and GFP fluorescence intensity was assessed.

Visual analysis of F_0_ individuals was performed using a MICROMED-3 fluorescence microscope equipped with an additional objective lens to facilitate non-invasive manipulation of specimens, including larvae and adults [47]. It should be noted that the *GMR-GAL4* driver, according to published data, is not strictly specific to eye imaginal discs [48,49]. Previous studies have demonstrated that *GMR-GAL4* induces expression of the reporter gene *lacZ* in wing and leg discs, the trachea, and the central nervous system. However, in our experimental system, using two independent *GMR-GAL4* lines and GFP fluorescence visualization (rather than X-Gal staining), we observed signal induction predominantly in ganglia, as well as in leg and wing discs, which is consistent with the findings of [49], indicating a broader expression pattern of this driver than previously assumed. The low level of background fluorescence in the control and its clear increase following irradiation suggest that this system can be used as a potential surrogate marker of radiation-induced cellular stress, even in the absence of canonical DNA damage-responsive elements within the driver construct.

### 2.3. Exposure to Ionizing Radiation

**Figure 4:**
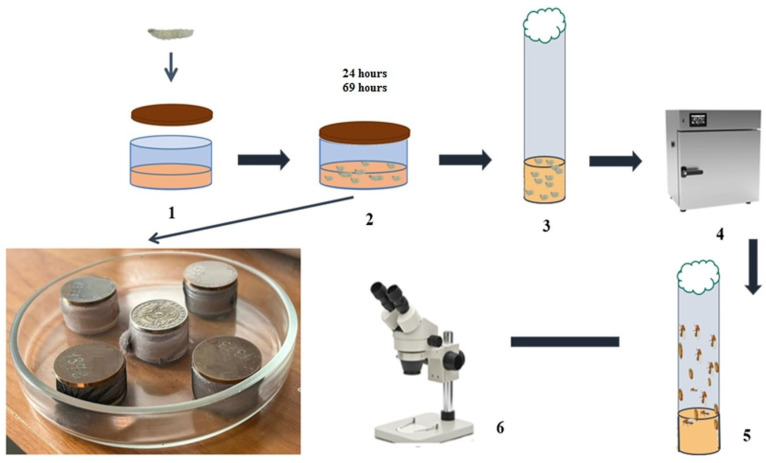
Experimental design of α-radiation exposure in *D. melanogaster* adults and larvae and the main experimental stages: (1) placement of adults and larvae into wells; (2) irradiation for 24–72 h; (3) transfer of irradiated adults or larvae into vials containing culture medium; (4) incubation; (5) adult emergence; (6) phenotypic analysis using a stereo microscope (MBS-10, ×2–7).

To assess the effects of α-radiation, both flies and larvae were irradiated using calibrated α-particle sources (described in detail in Table 1). Flies or first- and second-instar larvae (up to 50 individuals) were placed in wells containing a 1 mm-thick layer of standard medium (stage 2 in Figure 4). Irradiation was carried out using four sealed calibrated alpha sources with an energy spectrum characteristic of radon and its decay progeny (4.8–7.7 MeV), resulting in absorbed doses ranging from 1.90 to 44.96 mGy over exposure periods of 20–72 h. The irradiation setup is shown in Figure 4. Control groups were maintained under identical conditions in the same room without irradiation.

The sources were positioned directly above the surface of the culture medium at a fixed distance of 4 mm from the flies or larvae, ensuring minimal attenuation of alpha particles in the air gap. The diameter of the sources was 24 mm, and the active surface area was 16 ± 2 mm, which fully covered the exposed area of the wells (22 mm in diameter), ensuring uniform irradiation of the entire medium surface. The dose distribution across the sample area was calculated taking into account the source geometry and the solid angle of irradiation. Dose variation within the well did not exceed 15%, as confirmed by calculations using a point-source model within a solid angle of 2π steradians. The highest uniformity was achieved when the wells were centrally positioned relative to the source. To control for potential effects of microenvironmental factors (restricted air exchange, local heating), a dummy disc made of stainless steel, identical in geometry and mass to the alpha source but lacking radioactive coating, was placed over the control wells. Thus, the conditions for flies or larvae maintenance in the experimental and control groups differed only in the presence of ionizing radiation.

A key factor determining the biological response is not only the incident (input) dose but also the spatial distribution of absorbed energy within fly or larvae tissues. Considering that the range of alpha particles with energies of 4.8–7.7 MeV in biological tissue is approximately 30–80 μm, whereas the size of early-stage *D. melanogaster* larvae ranges from 150 to 300 μm in diameter (with a body length of 1–3 mm and a cuticle thickness of less than 10 μm), the alpha particle range is smaller than the larval body thickness. In adult *D. melanogaster*, alpha particles are likewise unable to penetrate the entire body of the insect during external irradiation; however, due to the relatively superficial localization of the gonads [50,51] in the abdominal region, they may reach peripheral tissues of the reproductive organs. For the estimation of tissue-specific absorbed doses in Equation (1), the source geometry factor (solid angle of 2π steradians) and the fraction of energy lost by particles in the food layer and air gap were taken into account. The calculations were based on the assumption that the entire kinetic energy of alpha particles entering the biological object is absorbed within the larval body. Thus, the mean absorbed dose in the tissues of flies and larvae was calculated as the ratio of the total energy deposited in all individuals within the vial to the total mass of their biological tissue. This approach enables appropriate extrapolation of the obtained relationships to other biological systems, taking into account differences in tissue geometry and target mass. The absorbed dose was calculated using the following equation:

(1) $$D=\frac{dE}{dm}\times t\times \frac{\pi r_{l}^{2}h_{l}\times N_{l}}{\pi r_{well}^{2}h_{f}},$$

where $\sum_{i=1}^{n}I_{i}\times E_{i}$ is the total energy of all incident particles (*I_i_*—the number of particles with energy *E_i_*); *r_l_* is the radius of the fly or larva (half of the body width); *h_l_* is the length of a fly or larva; *N_l_* —is the number of individuals in the well; *r_well_* is the radius of the well; *h_f_* is the height of the food layer in the well; *t* is the irradiation time (exposure duration). As follows from Equation (1), the absorbed dose was calculated taking into account the energy of alpha particles, the morphometric characteristics of the individuals, and the geometric parameters of the wells. The dose calculated using Equation (1) represents the whole-body average for the individual. Due to the short range of alpha particles, the actual absorbed dose in superficial target tissues may exceed this average.

Randomization was applied at several stages: (i) simple randomization; (ii) blocked (paired) randomization (fly or larvae for experimental and control groups were selected in parallel from multiple wells); and (iii) cluster randomization (random selection of varying numbers of fly or larvae from different vials).

### 2.4. Phenotypic and Statistical Analysis

Analysis of morphological abnormalities was performed in adult *D. melanogaster* using an MBS-10 stereomicroscope (×2–7). Changes in eye morphology, wing structure, bristle pattern, and body pigmentation were assessed. For each experimental group, at least 100 individuals were analyzed in three independent replicates. The sample size was determined in accordance with standard practices in *D. melanogaster* mutagenicity assays [45], based on practical feasibility under laboratory conditions and the availability of pilot data. Exclusion criteria were defined a priori. The following were excluded from the analysis: sterile males (in individual crosses), individuals that died before completion of the experiment, and larvae that failed to complete metamorphosis. The proportion of excluded individuals did not exceed 5% in any group. To minimize potential confounding factors, all control and experimental vials were placed in a single incubator in a fully randomized arrangement, thereby eliminating the influence of temperature, humidity, and light gradients. The irradiation sequence across experimental groups was also randomized. Additional stress factors (temperature fluctuations, disruption of circadian rhythms, and changes in culture medium or saline composition) were minimized through strict standardization of experimental conditions and media composition.

GFP fluorescence intensity in imaginal discs was quantified using ImageJ v1.54 software (NIH, Bethesda, MD, USA). Larvae were irradiated with calibrated alpha sources according to the protocol described in Figure 4 for 24–48 h at absorbed doses ranging from 2.28 to 29.97 mGy. Following irradiation, larvae were rinsed from the culture medium using Ephrussi–Beadle solution, dissected with fine laboratory needles, and imaginal discs were isolated. The discs were mounted on microscope slides in a drop of physiological saline and covered with coverslips. Fluorescence intensity was measured in both experimental and control groups (non-irradiated) using a MICROMED-3 fluorescence microscope. For each sample, the mean gray value was determined within a defined region of interest (ROI) after background subtraction. At least five imaginal discs per condition were analyzed. Comparisons were performed against control samples (non-irradiated but processed identically).

Statistical analysis was performed using StatPlus v7 and WINPEPI v11.65 software. Differences in the frequency of morphological abnormalities between experimental and control groups were assessed using the chi-square (*χ*^2^) test with Yates’ continuity correction [52]:

(2) $$\chi^{2}=\frac{{\left(\left|ad-bc\right|-\frac{N}{2}\right)}^{2}N}{\left(a+b\right)\left(c+d\right)\left(a+c\right)\left(b+d\right)},$$

where *a*, *c*—represent flies without morphological abnormalities in the experimental and control groups, respectively; *b*, *d*—represent flies with morphological abnormalities in the experimental and control groups; and *N* is the total number of flies. Differences were considered statistically significant at p<0.05 and p<0.01. The relationship between absorbed dose and the frequency of morphological abnormalities induction was evaluated using Pearson’s correlation coefficient. The significance of the correlation coefficient was assessed using Student’s *t*-test [52,53].

## 3. Results

Five experimental series were conducted to evaluate the effects of alpha radiation on *D. melanogaster* using the following test systems: the *Muller-5*, systems with attached X chromosomes, systems with simultaneously attached X-X and X-Y chromosomes, and a GFP-based fluorescent reporter system. Irradiation was performed using calibrated alpha sources according to the protocol described in Section 2.3, at absorbed doses ranging from 1.90 to 44.96 mGy. Statistical analyses of radiation-induced effects, assessed by the frequency of morphological abnormalities, are presented below.

### 3.1. Muller-5 (Basc) Test System

Following alpha irradiation in the *Muller-5 (Basc)* test system within the studied dose range, the following morphological abnormalities were identified in F_2_ progeny that inherited the irradiated X chromosome: black spots on the body, abdomen, and head (melanotic tumors), unexpanded wings, abnormalities in wing venation, wing blisters, partial absence of the thorax, absence of the wing and haltere, and general body deformities. These abnormalities are observed in individuals that were not directly irradiated but inherited an irradiated X chromosome from the father, indicating heritable alterations in germline cells. Statistical analysis of the *Muller-5* experiment after 24 h exposure revealed statistically significant differences between the experimental and control groups. The results of the statistical analysis are presented in Table 2.

As shown in Table 2, the experimental χ^2^ value was 12.50, exceeding the critical value (χ^2^_critical_ = 3.84) at one degree of freedom (df = 1) and a significance level of *p* < 0.05. Since a significance level of 0.05 is generally accepted as the minimum threshold in biomedical research, the observed χ^2^ excess indicates statistically significant differences between the experimental and control groups. The obtained results indicate that alpha radiation induces a statistically significant increase in the frequency of morphological abnormalities in the offspring of irradiated adult flies. This suggests heritable alterations in the genetic background (mutations or epimutations), which compromise developmental stability and increase the likelihood of morphoses in the offspring. Morphoses themselves are not inherited; however, susceptibility to their occurrence may be transmitted across generations, which is consistent with the concept of genetic control of developmental canalization [54]. To further validate these findings and to assess the probability of morphosis induction with higher sensitivity, additional experiments were conducted using short-term genetic test systems.

### 3.2. Sex-Linked Chromosome Test Systems

**Figure 5:**
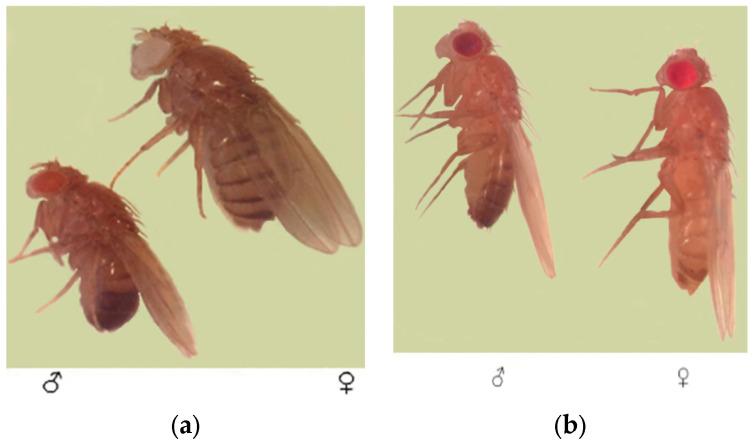
Genetic tester lines of *D. melanogaster* used in the experiment: (**a**) *1-112*; (**b**) *EP-2*.

To verify the mutagenic effects observed in the *Muller-5* assay and to investigate potential epigenetic effects of alpha-particle exposure (particularly those generated by radon isotopes), more sensitive short-term test systems were employed. These included *D. melanogaster* lines with attached X chromosomes (*1-112*) and a system with attached X-X and X-Y chromosomes (*EP-2*) (Figure 5). Larval exposure durations ranged from 20 to 72 h. The experiments were designed to assess epigenetic responses induced by alpha particles, which, under environmental conditions, are predominantly generated by radon and its decay progeny.

In the first series of experiments with 20 and 24 h exposures (source No. 1), the frequency of morphosis induction was evaluated in two tester lines. Here, abnormalities are recorded in adult flies that developed from irradiated larvae, indicating somatic damage. The results of statistical analysis at an absorbed dose of 2.28 mGy are presented in Table 3.

The data presented in Table 3 demonstrate a statistically significant increase in the frequency of morphological abnormalities in both test systems. In the *EP-2* line, the experimental χ^2^ value was 24.78 (*p* < 0.01), while in the 1-112 line it was 8.55 (*p* < 0.05), confirming the teratogenic effect of alpha radiation.

To assess the heritability of induced changes, females of the *EP-2* and *1-112* lines were crossed with irradiated males of the wild-type (*Oregon-R*) line. In these crosses, any abnormalities observed in the F_1_ progeny are attributable to mutagenic damage in the paternal germline. The analysis of the F_1_ progeny from these crosses following 24 h exposure using different sources is presented in Table 4 and Table 5.

In all experimental F_1_ groups, a statistically significant (*p* < 0.05 and *p* < 0.01) increase in the frequency of morphoses compared to controls was observed. The highest frequency of morphoses was recorded in the *EP-2* test system (up to 4.20%), indicating its high sensitivity to alpha irradiation. With an increase in exposure duration to 72 h, a different pattern was observed in the test system with the attached X chromosome test system (*1-112*) (Table 6).

In contrast to the 24 h exposure, no statistically significant increase in the frequency of morphoses was observed after 72 h of irradiation with sources No. 2 and No. 1 (*p* > 0.05). However, exposure to sources No. 4 and No. 3 resulted in a significant increase in the frequency of morphoses, reaching 4.68% (*p* < 0.05) and 7.60% (*p* < 0.01), respectively.

**Figure 6:**
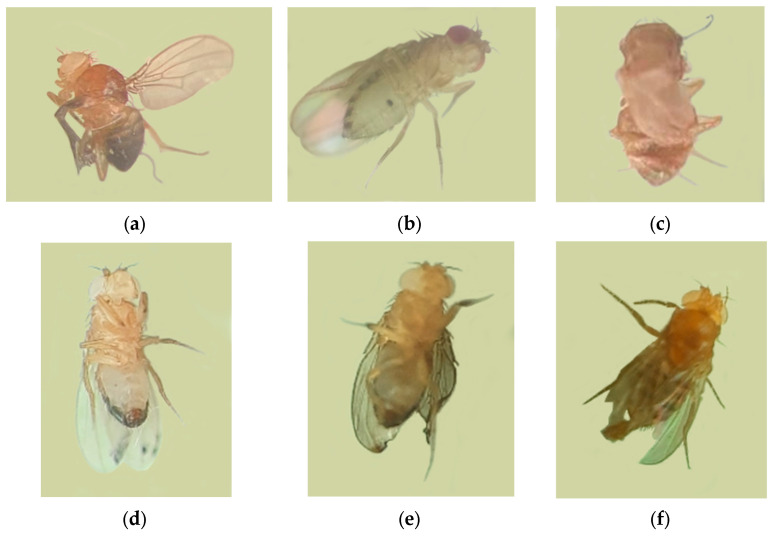
Morphological abnormalities observed in F_1_ and F_2_ *D. melanogaster* in the attached X chromosome test system crossed with *Oregon-R* males: (**a**) F_1_ twisted and partially collapsed wings; (**b**) F_1_ altered thorax and leg pigmentation (“glassy” phenotype); (**c**) F_1_ tergite abnormalities; (**d**) F_1_ absence of one wing with thoracic deformation; (**e**) F_1_ abdominal melanoma; (**f**) F_2_ shortened right wing.

The spectrum of morphological abnormalities identified in F_1_ and F_2_ adults was broad and included melanotic formations (Figure 6): “generalized melanomas” on the abdomen and thorax; twisted and underdeveloped (shortened) wings; blister-like wing deformations or complete wing loss accompanied by thoracic malformation; abnormalities in tergite structure and wing venation; as well as changes in limb pigmentation and the ommatidial structure of the eyes.

**Figure 7:**
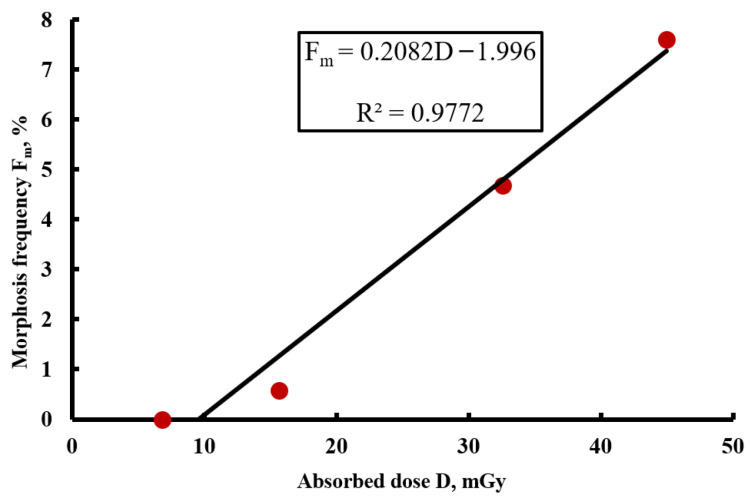
Dose–response relationship between absorbed dose of α-radiation and the frequency of morphoses in *D. melanogaster* following 72 h exposure.

To evaluate the dose–response relationship, the experimental data were grouped according to absorbed dose, regardless of the radiation source type. The analysis showed that, at 24 h exposure, no significant correlation between absorbed dose and the frequency of morphoses was detected, likely due to insufficient time for the manifestation of latent damage under chronic irradiation conditions. However, when the exposure duration was increased to 72 h, a clear positive correlation was observed. The results of the analysis of frequency of morphoses in the F_1_ generation as a function of absorbed dose (based on Table 6 data) are presented in Figure 7.

As shown in Figure 7, a strong positive correlation was observed between absorbed dose and the frequency of morphoses, with a Pearson correlation coefficient of r = 0.98 (*p* < 0.01). As the dose increased from 6.84 mGy to 44.96 mGy, the frequency of morphoses rose from 0% to 7.6%, indicating a pronounced dose-dependent effect under prolonged (72 h) exposure. The absence of a clear dose–response relationship at 24 h exposure may suggest a threshold-like manifestation of radiation-induced damage or the requirement for longer exposure to accumulate a critical number of biological events.

Thus, statistical analysis using the χ^2^ test confirmed statistically significant differences between experimental and control groups (*p* < 0.05 and *p* < 0.01) for most of the studied groups. The obtained results demonstrate that alpha radiation within the studied low-dose range exerts pronounced mutagenic and teratogenic effects, the magnitude of which depends on both the genetic system and the duration of exposure.

### 3.3. Reporter Gene of the UAS-GFP System

**Figure 8:**
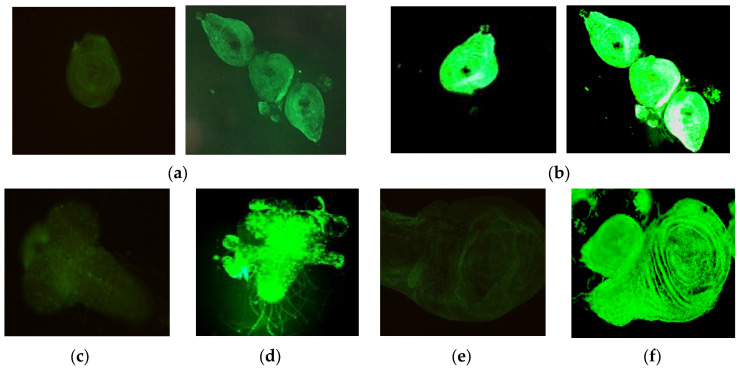
Visualization of fluorescence in imaginal discs of *D. melanogaster* following 24 h α-irradiation of larvae: (**a**,**b**) Leg imaginal disc: (**a**) control (non-irradiated); (**b**) experimental (dose 14.99 mGy), showing intense fluorescence. (**c**,**d**) Ganglion: (**c**) control; (**d**) experimental (dose 10.41 mGy). (**e**,**f**) Wing imaginal disc: (**e**) control; (**f**) experimental (dose 14.99 mGy).

To visualize and quantitatively assess the teratogenic effects of α-radiation, the *GAL4/UAS-GFP* reporter system was employed. The principle of this method is based on the assumption that activation of green fluorescent protein (GFP) expression occurs in response to DNA damage and cellular stress signals of imaginal discs and histoblast cells, which are precursor tissues of the adult fly. Irradiation of larvae carrying the genetic constructs *GMR-GAL4* and *UAS-GFP* resulted in the induction of GFP expression in multiple imaginal discs. Representative images demonstrating increased fluorescence intensity in experimental groups compared to controls are shown in Figure 8.

As shown in Figure 8, fluorescence in control samples (a, c, e) remained at background levels. The level of background fluorescence in the control was consistently low across all experimental replicates, confirming the absence of significant tissue autofluorescence and enabling reliable detection of the induced signal. Exposure to α-particles induced distinct fluorescence signals in cells of leg imaginal discs (b), ganglia (d), and wing discs (f), indicating activation of the stress response.

For quantitative evaluation, larvae were irradiated within a dose range of 2.28 to 29.97 mGy with exposure durations of 24–48 h. Following the dissection of imaginal discs and measurement of fluorescence intensity using ImageJ software (as described in Section 2.4), a comparative analysis between experimental and control samples was performed. Dose–response analysis revealed a threshold-like pattern of fluorescence induction. At absorbed doses below 10 mGy, no statistically significant increase in fluorescence intensity compared to the controls was detected. However, above this threshold (within the range of 10–15 mGy and higher), a marked increase in GFP expression was observed, correlating with increasing absorbed dose. The mean fluorescence intensity increased approximately 2.5-fold. This indicates the presence of a dose threshold for the activation of detectable stress responses under conditions of chronic α-irradiation. To exclude artifacts associated with possible radiation-induced tissue autofluorescence, control samples were processed identically to the experimental ones (including dissection and imaging procedures). In the control groups, fluorescence consistently remained at background levels. Moreover, GFP induction was observed in the same tissues (ganglia and leg imaginal discs) previously described by Li et al. [49] for *GMR-GAL4*—mediated expression, confirming the specificity of the signal and excluding its random nature.

Thus, the observed increase in fluorescence intensity in sensitive tissues (imaginal discs of legs, wings, antennae, and histoblasts) under α-particle exposure serves as indirect evidence of cellular stress induction and possible DNA damage. The obtained data demonstrate that α-radiation, within the studied dose range, exhibits pronounced teratogenic effects, as detected using this surrogate marker system.

## 4. Discussion

The present study provides a comprehensive assessment of the mutagenic and teratogenic effects of α-radiation (4.8–7.7 MeV) in the low-dose range (1.90–44.96 mGy) using *D. melanogaster* as a model organism and three independent genetic approaches: the classical *Muller-5* test, short-term sex chromosome-linked test systems, and the *GAL4/UAS-GFP* reporter system with quantitative fluorescence analysis. For the first time within this dose range, a linear dose–response relationship for the induction of morphoses in offspring (r = 0.98 at 72 h exposure) and a threshold-like activation of GFP fluorescence (>10 mGy) were demonstrated, indicating the mutagenic potential of α-particles even under low-intensity chronic exposure and supporting the presence of teratogenic effects.

The results obtained using the classical *Muller-5* test revealed a statistically significant increase in the frequency of morphoses (χ^2^ = 12.50, *p* < 0.05) in the second generation of flies. The observed spectrum of abnormalities (melanotic masses, wing deformities, and thoracic segmentation defects) is characteristic of damage induced by high linear energy transfer (LET) radiation, consistent with previous reports [55,56]. Earlier studies by our research group have shown that melanotic formations in *D. melanogaster* arise in response to various mutagenic factors and can serve as markers of radiation-induced genetic instability [44,57]. The formation of melanotic masses, associated with immune dysregulation and hemocyte proliferation, further supports the suitability of this model for studying radiation-induced alterations resembling inflammation-mediated carcinogenesis [18,19,58,59].

Additional evidence of the mutagenic activity of α-radiation was obtained using sex chromosome-linked test systems, including attached X chromosome systems and combined X-X/X-Y configurations. The frequency of morphoses in F_1_ progeny reached 4.20% in experimental groups compared to 0.39% in controls (χ^2^ = 45.03, *p* < 0.01 in the EP-2 line). Notably, the manifestation of abnormalities across F_2_ generations observed in this study may be associated with maternal effects of radiosensitivity-related genes. Previous studies [60] have demonstrated a prolonged maternal effect of the *rad201* gene in *D. melanogaster*, conferring increased radiosensitivity across multiple generations following irradiation. As reported elsewhere [61,62], such abnormalities may result from pleiotropic effects of damaged regulatory genes and radiation-induced chromatin instability.

A key finding of this study is the strong positive correlation (r = 0.98) between absorbed dose and the frequency of morphoses following 72 h exposure (Figure 7). Increasing the dose from 6.84 to 44.96 mGy resulted in an increase in morphosis frequency from 0% to 7.6%. The absence of a clear dose–response relationship at 24 h exposure suggests a threshold-like manifestation of radiation-induced damage or the requirement for prolonged exposure to accumulate a critical number of biological events, in line with ongoing discussions regarding the applicability of the linear no-threshold (LNT) model at low doses [36,37]. As highlighted by Wilson in a recent review [63], the applicability of the LNT model below 100 mGy remains controversial, with evidence supporting both linear and threshold/hormetic responses.

The application of the *GAL4/UAS-GFP* reporter system enabled a transition from purely phenotypic observations to a surrogate analysis of cellular stress responses. Our data indicate a broader domain of *UAS-GFP* expression following α-irradiation, detected in several larval tissues, including ganglia, leg imaginal discs, and wing discs. Although this driver does not inherently contain canonical stress-inducible elements, the observed dose-dependent and tissue-specific increase in fluorescence may reflect activation of the cellular stress associated with radiation-induced damage. This approach is considered here as a screening-level surrogate method, requiring further validation using dedicated DNA damage-responsive reporters. Quantitative fluorescence analysis using ImageJ revealed a threshold-like activation of GFP expression: no significant increase was observed below 10 mGy, whereas doses above this threshold (10–15 mGy and higher) resulted in a 2.5-fold increase in fluorescence intensity. This indicates the presence of a dose threshold for activation of detectable stress responses under conditions of chronic α-irradiation. Activation of signaling pathways regulating morphogenesis and stress responses has been described previously [64], including the involvement of hydrocarbon receptor homologs and nucleosome assembly proteins in developmental regulation, memory, and detoxification processes. These findings are consistent with the teratogenic developmental abnormalities observed in this study. Furthermore, studies on radiation-induced tissue regeneration [65] highlight the roles of Ras/MAPK, JAK/STAT, Hippo, Wingless, and Nurf-38 signaling pathways in controlling tissue recovery, which may explain the variability of morphological abnormalities observed. The present results are also consistent with previous findings on DNA repair induction and stress-responsive gene expression in *D. melanogaster* [66,67].

The α-sources used in this study (4.8–7.7 MeV) simulate exposure to natural radon isotopes and their decay products (^218^Po, ^214^Po), providing practical relevance for regions with elevated natural radiation background, such as Kazakhstan. The observed ability of α-particles to induce dose-dependent genetic damage even at low doses (1.90–44.96 mGy), as well as teratogenic effects, underscores the need for improved radiation safety measures and public awareness. The high sensitivity of the *GAL4/UAS-GFP* system also suggests its potential application in the development of biological dosimeters for monitoring radon-prone areas.

Several limitations should be considered. First, despite approximately 75% homology with human disease-related genes [26], *D. melanogaster* is an invertebrate model with physiological and DNA repair differences compared to mammals. Therefore, observed morphoses should be interpreted as indicators of mutagenesis rather than direct evidence of carcinogenesis in humans. Second, the employed test systems primarily detect large-scale morphological alterations; whereas detailed analysis of point mutations and underlying molecular mechanisms requires additional approaches such as sequencing and gene expression profiling. Third, extrapolation of dose–response relationships to humans should be performed with caution due to differences in tissue geometry and organ size, although the proposed methodology for tissue-specific dose estimation provides a conceptual framework for such comparisons.

Overall, the results are consistent with evidence demonstrating the higher effectiveness of α-particles and neutrons compared to γ-radiation in inducing chromosomal aberrations such as inversions and translocations [68]. Recent studies in *D. melanogaster* have also shown that DNA damage responses depend on both dose rate and radiation quality [69]. The observed abnormalities may be interpreted as complex mutation-like events affecting morphogenetic pathways. The increase in the fluorescence intensity and disturbances in morphogenesis in the *GAL4/UAS-GFP* system are consistent with studies on the role of potassium channels (Irk) and BMP/Dpp signaling pathways in *D. melanogaster* wing development. Future research should focus on: (i) molecular validation of the observed effects using RNA sequencing and analysis of DNA repair gene expression (e.g., *BRCA1/2* and *ATM* homologs); (ii) investigation of transgenerational effects across multiple generations (F_2_–F_4_); and (iii) development of *GAL4/UAS-GFP*-based biosensor systems for monitoring radon-prone environments.

In summary, the present study, employing quantitative statistical approaches (χ^2^ test and Pearson correlation analysis), demonstrates that α-radiation in the low-dose range (up to 44.96 mGy) exerts pronounced mutagenic and teratogenic effects, dependent on both genetic background and exposure duration. The observed dose–response relationship at 72 h exposure (r = 0.98) and threshold-like increase in the fluorescence (>10 mGy) contribute to the ongoing discussion regarding the applicability of the LNT model for low-dose α-radiation and support the use of *D. melanogaster* as a sensitive system for ecogenetic monitoring of radiation-prone environments.

## 5. Conclusions

In the present study, a comprehensive quantitative assessment of the mutagenic and teratogenic effects of α-radiation in the low-dose range (1.90–44.96 mGy) was performed for the first time using the *D. melanogaster* model. Using three independent genetic approaches, it was demonstrated that α-particles with energies of 4.8–7.7 MeV, associated with radon and its decay progeny, induce a dose-dependent increase in the frequency of morphological abnormalities both in directly irradiated individuals and in their offspring, as well as an increase in the fluorescent signal in the *GAL4/UAS-GFP* reporter system in sensitive tissues. The observed linear relationship at the level of morphogenesis in the offspring and the threshold activation of cellular stress signals indicate the complex nature of the biological effects of low-intensity chronic irradiation. Notably, in the *GAL4/UAS-GFP* reporter system, increased fluorescence was observed only at doses above 10 mGy (threshold effect), whereas among the genetic assays employed, the highest sensitivity was exhibited by the sex-linked chromosome test (*EP-2* line). These findings support the use of *D. melanogaster* as a sensitive test system for biological dosimetry in radon-prone areas and contribute to the ongoing discussion on the applicability of the linear no-threshold model for assessing the risks of α-radiation.

## Figures and Tables

**Table 1:** Characteristics of the alpha sources used in the experiments.

Source No.	Radionuclide Activity in Source, kBq	Alpha-Particle Energy at *p* = 0.95, keV	Alpha-Particle Flux Through the Active Surface of the Source into a Solid Angle of 2π Steradians (sr), [s^−1^]
No. 1	40.1	5499 ± 3	1.99 × 10^4^
No. 2	3.80	5156 ± 5	1.88 × 10^3^
No. 3	30.6	4824 ± 6; 5499 ± 6; 5156 ± 6	1.89 × 10^4^
No. 4	38.6	4750 ± 8; 5455 ± 8; 5968 ± 8; 7653 ± 8	1.54 × 10^4^

**Table 2:** Results of the statistical analysis of mutagenic effects following irradiation of adult *D. melanogaster* with source No. 1 at 24 h exposure in the *Muller-5* test system.

Experimental	*a* (flies without morphological abnormalities)	*b* (flies with morphological abnormalities)	⅀	$\chi^{2}$ Value
	3796	32	3828	12.50 *
Control	*c* (flies without morphological abnormalities)	*d* (flies with morphological abnormalities)		
	3688	8	3696	
⅀	7484	40	7524	

Note. * *p* < 0.05 compared to control (χ^2^_critical_ = 3.84, df = 1).

**Table 3:** Results of the statistical analysis of the teratogenic effect in short-term test systems *EP-2* and *1-112* following irradiation of larvae with source No. 1 under a 24 h exposure.

Test-System	*EP-2*			*1-112*		
Experimental	*a* (flies without morphological abnormalities)	*b* (flies with morphological abnormalities)	⅀	*a* (flies without morphological abnormalities)	*b* (flies with morphological abnormalities)	⅀
	2143	46	2189	3258	57	3315
Control	*c* (flies without morphological abnormalities)	*d* (flies with morphological abnormalities)		*c* (flies without morphological abnormalities)	*d* (flies with morphological abnormalities)	
	2129	8	2137	1379	8	1387
⅀	4272	54	4326	4637	65	4702
$\chi^{2}$ value	24.78 **			8.55 **		

Note. ** *p* < 0.01; statistically significant differences between the experimental and control groups (χ^2^_critical_ = 6.64, df = 1).

**Table 4:** Results of the statistical analysis of the mutagenic effect in F1 adults obtained from crosses between females of the *1-112* test system and *Oregon-R* males following 24 h exposure.

Group (Source No.)	*a* (Flies Without Morphological Abnormalities)	*b* (Flies with Morphological Abnormalities)	⅀	Morphological Abnormality Frequency (%)	$\chi^{2}$ Value
Experimental (No. 2)	1311	30	1341	2.24	4.15 *
Experimental (No. 1)	412	10	422	2.37	6.18 *
Experimental (No. 3)	279	11	290	3.79	8.58 **
Control	*c* (flies without morphological abnormalities)	*d* (flies with morphological abnormalities)			
	614	5	619	0.81	

Note. * *p* < 0.05 (χ^2^_critical_ = 3.84, df = 1); ** *p* < 0.01 (χ^2^_critical_ = 6.64, df = 1).

**Table 5:** Results of the statistical analysis of the mutagenic effect in F_1_ adults obtained from crosses between females of the *EP-2* test system and *Oregon-R* males following a 24 h exposure.

Group (Source No.)	*a* (Flies Without Morphological Abnormalities)	*b* (Flies with Morphological Abnormalities)	⅀	Morphological Abnormality Frequency (%)	$\chi^{2}$ Value
Experimental (No. 2)	666	28	694	4.20	45.03 **
Experimental (No. 1)	1797	38	1835	2.11	19.44 **
Experimental (No. 3)	657	24	681	3.65	36.47 **
Control	*c* (flies without morphological abnormalities)	*d* (flies with morphological abnormalities)			
	1778	7	1785	0.39	

Note. ** *p* < 0.01; (χ^2^_critical_ = 6.64, df = 1).

**Table 6:** Results of the statistical analysis of the mutagenic effect in F_1_ hybrid adults obtained from crosses between females of the X-linked test system (*1-112*) and males (*wa*) following a 72 h exposure.

Group (Source No.)	*a* (Flies Without Morphological Abnormalities)	*b* (Flies with Morphological Abnormalities)	⅀	Morphological Abnormality Frequency (%)	$\chi^{2}$ Value
Experimental (No. 2)	78	0	78	0	0
Experimental (No. 1)	172	1	173	0.57	0.015
Experimental (No. 4)	122	6	128	4.68	4.58 *
Experimental (No. 3)	254	21	275	7.6	9.43 **
Control	*c* (flies without morphological abnormalities)	*d* (flies with morphological abnormalities)			
	136	0	136		

Note. * *p* < 0.05 (χ^2^_critical_ = 3.84, df = 1); ** *p* < 0.01 (χ^2^_critical_ = 6.64, df = 1).

## Data Availability

The data presented in this study are available upon request from the corresponding author, Yuliya Zaripova.
